# Editorial Notes

**Published:** 1936

**Authors:** 


### Editorial Notes

**Accession of King George VI.**

The abdication of H.M. King Edward VIII on 12th December, after a reign lasting only 325 days, has brought his brother the Duke of York to the throne with the style of King George VI. The circumstances which led the Duke of Windsor to relinquish the burden of sovereignty were sympathetically related by the Prime Minister (Mr. Baldwin) in Parliament, and by our former king in his farewell broadcast. The only feeling that need be expressed is one of heartfelt gratitude that the Duke of York should have consented to face the task from which King Edward VIII. shrank, and has accepted the heavy and often thankless office of King of Great Britain and the Dominions and Emperor of India. All his subjects welcome King George with loyal affection, wishing him and our new Queen Elizabeth and the Princesses Elizabeth and Margaret Rose long life and happiness.

**Bristol University Expedition to Central Asia.**

Through the generosity of Miss Hilda Wills an expedition under the auspices of the University is being planned to explore the buried cities of the Tarim Basin in Chinese Turkestan. The leader of the expedition is to be Mr. Evert Barger, Lecturer in Mediæval History. Mr. Miles Burkitt of Cambridge will be the Chief

Archæologist. The expedition will survey the cities previously discovered by Sir Aurel Stein, Sir Sven Hedin and Professor Lecoq, as far as the Oasis of Charchard, and will endeavour to find new sites suitable for future excavations. It is intended to make this a preliminary survey, to pave the way for a larger expedition if financial support can be obtained at some future time. The enterprise will be followed with keen interest, and the University is to be heartily congratulated on its association with such a project.

**Social Survey of Bristol.**

THE Report of the Colston Research Society for 1935–36 contains the announcement that a fund has been established for carrying out a Social Survey of Bristol. The sum of £1,662 5s. 0d. has already been collected, and Mr. Herbert Tout, M.A., has been appointed to a Colston Research Fellowship in the University for the purpose of directing the Survey. Mr. Tout comes to this work with experience in economic investigation gained elsewhere, more particularly the widely-known inquiries of the Employment Stabilization Research Institute of the University of Minnesota. The Survey in Bristol is intended to study not only poverty and its causes, there are also social problems of those who live above the poverty line which will be investigated. The operation of what are known as the "Social Services" is to be a new and important object of the Survey. An attempt is to be made to discover the extent to which the various services are used by members of different occupation and income classes, to discover what gaps have been left in the piecemeal growth of particular services, and how far local administration has bridged the gaps and fostered co-operation. In such a survey the medical services must play a substantial part. Fundamentally, the public weal is based on sound mental and physical health. The *common* wealth is not to be measured in terms of property or money.

**New Faculty of Medicine Buildings and Dental Hospital.**

AT the Annual Meeting of the University Court it was reported that, owing to the lack of accommodation for the pre-clinical subjects, it had again been necessary to limit the number of students admitted to the Faculty of Medicine, and that Council had approved a proposal for the erection of a new building to house all departments of the Medical School. Whilst plans for the entire building will shortly be obtained, the funds at the disposal of the University will not cover the cost, and it is anticipated that the first step will be to provide for the needs of Anatomy and Physiology.

It was also reported that property had been bought by the University which would shortly be adapted to serve as a Dental School. This School will be in close proximity to the Bristol Royal Infirmary.

These developments in the University will cause general satisfaction, and we hope that funds will soon be forthcoming to extend the new buildings of the Faculty of Medicine to include the Clinical Departments. The segregation of these departments in Canynge Hall at a long distance from Anatomy, Physiology, the Medical Library, and the Hospitals is an acknowledged makeshift which needs to be remedied.

**Centenary of Leopold Von Schrötter's Birth.**

On 30th January the University of Vienna and the Vienna Laryngo-Rhinological Society will celebrate the hundredth birthday of Leopold Von Schrötter. Born on 5th February, 1837, he was one of the great pioneers of laryngology, and his researches and methods still influence present practice. He was the founder and first chief of the Vienna University Laryngological Clinic, the first such special clinic in the world. He is remembered for his work on laryngeal stenosis and as one of the founders of the modern campaign against tuberculosis. Near Vienna, in Alland, he founded the first climatic-dietetic establishment for tuberculous patients.

To mark the occasion a marble tablet, with a medallion of Schrötter by Professor Von Zumbush, will be unveiled in the Arkadenhof of the University of Vienna: speeches will be made by Professor Arzt (Rector of the University), Professor Karl (Dean of the Medical Faculty), Professor Sorgo (formerly pupil of Schrötter), and Professor Marschik (President of the Vienna Laryngo-Rhinological Society).

